# Effect of cold atmospheric pressure plasma on wettability of implant prosthetic materials: an in-vitro study

**DOI:** 10.1186/s40729-026-00685-3

**Published:** 2026-05-04

**Authors:** Moataz Bayadse, Leonie Grander, Lisa Steiner, Samir Abou-Ayash, Stefan Wentaschek

**Affiliations:** 1 Department of Prosthodontics and Material Science, University Medical Center Mainz, Augustusplatz 2, 55131 Mainz, Germany; 2 Department of Reconstructive Dentistry and Gerodontology, School of Dental Medicine, University of Bern, Freiburgstrasse 7, 3007 Bern, Switzerland

**Keywords:** Cold atmospheric pressure plasma, Surface treatment, Titanium, Zirconia, Wettability, Contact angle, Abutment crown, CAD-CAM

## Abstract

**Purpose:**

Surface wettability is a key factor in bonding of dental materials. The aim of the present study was to investigate the influence of different exposure times of cold atmospheric pressure plasma at constant power on the surface wettability of various dental materials employed in implantology, using the “Piezo-Brush^®^” PZ3 cold atmospheric pressure plasma device.

**Methods:**

Seventy-five standardized specimens made of titanium alloy, zirconia, lithium disilicate and polymer-infiltrated hybrid ceramic network were manufactured and polished. Specimens of each material were divided into 5 groups (each *n* = 15). Four groups were cold atmospheric pressure plasma treated for 5, 10, 20, 30 s and compared to the untreated control group. Surface wettability was assessed by measuring the contact angle of distilled water using a goniometer. Statistical analysis was performed using one-way ANOVA.

**Results:**

The mean contact angle was significantly reduced by 60–80% after 5 s of cold atmospheric pressure plasma treatment for all investigated materials (*p* < 0.001). Prolonged cold atmospheric pressure plasma exposure resulted in further reduction in mean contact angle values for titanium at each additional treatment duration (*p* < 0.001), whereas for lithium disilicate a reduction was observed only up to 10 s. For zirconia and polymer-infiltrated hybrid ceramic network, no further reduction in contact angle was observed beyond 5 s of cold atmospheric pressure plasma treatment.

**Conclusion:**

Cold atmospheric pressure plasma treatment significantly increases surface wettability after short exposure times. Within the limitations of this study, these findings may indicate a potential improvement in adhesion behavior. However, under the plasma parameters applied, extending treatment beyond 5 s for zirconia and polymer-infiltrated hybrid ceramic network or beyond 10 s for titanium and lithium disilicate did not result in further reduction of contact angle. The clinical relevance of these findings requires further investigation.

**Graphical abstract:**

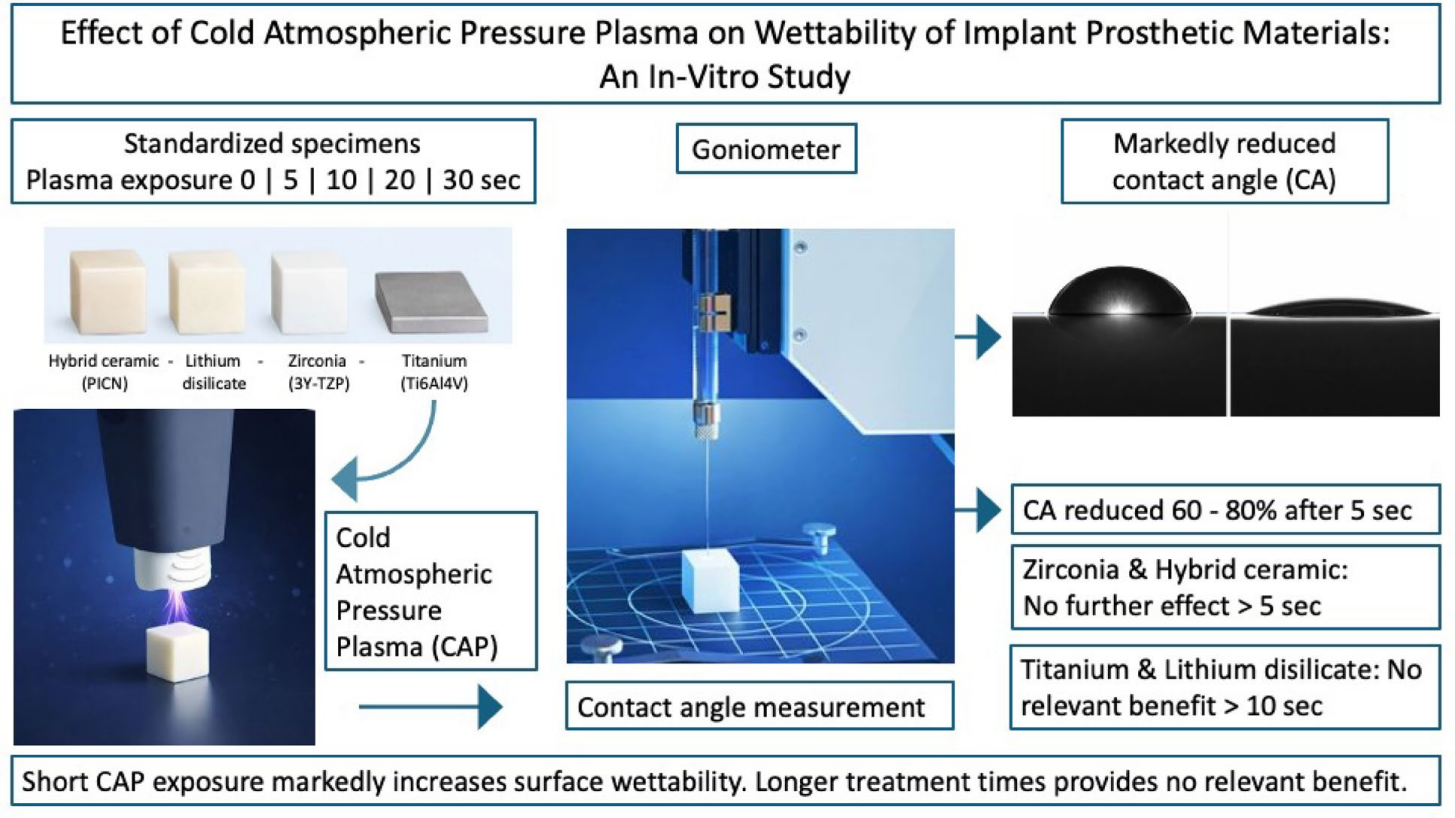

## Background

Adhesive bonding between different materials is a daily necessity in every field of dentistry. Materials such as ceramics or metals need to be bonded to each other or to the tooth structure. Due to different chemical, physical and mechanical properties, each material has a specific pre-treatment protocol. These pre-treatments in combination with suitable adhesives are necessary to achieve a durable and stable bond [1]. Another important factor is the elimination of contamination, which could prohibit the wetting of the luting material and reduce the bond strength [2].

Wettability is a key prerequisite for effective adhesive bonding, as it determines the ability of a luting material to spread over and interact with the substrate surface. Adequate wetting enables intimate contact at the bonding interface, supporting micromechanical interlocking and chemical interactions. Wettability is commonly assessed by the contact angle, with lower values indicating improved wetting behaviour. Insufficient wettability may result in incomplete surface coverage and reduced bond strength [3].

Pre-treatment steps to improve the adhesive bond are methods that promote micromechanical retention, wetting, chemical bonding or all of them [4–8]. Common pretreatment protocols for titanium and zirconia involve sandblasting followed by the application of a phosphate primer, whereas the gold standard for lithium disilicate ceramics is etching with hydrofluoric acid (HF) before primer application [4–12]. Due to the increasing use of digital workflows in restorative dentistry, the bonding of CAD/CAM-fabricated ceramic crowns to prefabricated titanium bases (Ti-bases) has become common clinical practice [6–8, 13]. Various studies have shown that bonding CAD/CAM-manufactured crowns after conditioning with the recommended protocols results in sufficient bond strength [14, 15]. However, debonding, especially of CAD/CAM hybrid crowns occurs in clinical practice. Furthermore, conventional pretreatment methods can be associated with several problems. Sandblasting with Al_2_O_3_ powder can lead to microcracks in the ceramic and HF can cause health problems during processing [16]. The differences of pretreatment protocols, for example regarding sandblasting pressure or exposure times of chemicals can lead to processing errors in clinical practice and may result in premature failure of the restoration [17].

Cold atmospheric pressure plasma (CAP) is already established in automotive industry or aviation engineering and is intended to improve the surface characteristics for adhesive bonding [18]. Several studies have investigated the influence of CAP on dental materials [19], and its use for decontamination [20, 21]. Plasma systems applicable in dentistry differ in their generating methods, operating temperature and pressure, and process gas. Some systems operate in ambient air and do not require vacuum equipment or inert gas supply. This offers practical advantages by enabling simple and efficient integration into routine dental laboratory workflows.

CAP generated with ambient air has been investigated for conditioning implant-prosthetic materials. In previous studies the expected positive effects on bond strength could not be confirmed. It was even reported that CAP treatment, whether used alone or in combination with components of conventional pretreatment protocols, not only failed to show improvement but even resulted in a decline of bond strength [22, 23]. The reasons for the lack of enhanced bond strength remain unclear. One potential explanation discussed in the literature is the insufficient specification and standardization of plasma process parameters such as exposure time and power settings for the effective conditioning of dental materials.

Therefore, the aim of the present study was to investigate the effect of different CAP exposure times at constant power on the wettability of various materials commonly used in implant prosthodontics. The null hypothesis was that cold atmospheric pressure plasma treatment would not affect the wettability measured by the contact angle (CA).

## Methods

### Preparation of specimens

Seventy-five platelets (20 mm x 20 mm x 5 mm) were cut out of a titanium sheet (Ti6Al4V, Elli ASTM 136 F, HWN titan GmbH, Mönchengladbach, Germany). These were manually polished in two stages using pumice powder followed by a polishing brush. After polishing, the specimens were cleaned using alcohol and distilled water. In addition, 75 specimens each were fabricated from three different ceramics: zirconia 3Y-TZP (IPS e.max ZirCAD LT, Ivoclar Vivadent, Ellwangen, Germany), lithium disilicate (IPS e.max CAD, Ivoclar Vivadent, Ellwangen, Germany) and a polymer-infiltrated hybrid ceramic network (PICN) (ENAMIC^®^, VITA-Zahnfabrik, Bad Säckingen, Germany) using subtractive CAD/CAM milling. The samples were designed as cubes (10 mm x 10 mm x 10 mm). The zirconia samples were sintered in the Programat S1 furnace and the lithium disilicate samples were crystallized in the Programat EP 5010 furnace according to the manufacturer’s instructions. All samples were then polished using diamond grinding pads with grit sizes of 50, 100, 200, 400 and 800 grit under water cooling and cleaned with alcohol and distilled water. The surface roughness was evaluated using a perthometer (Pethen Perthometer PRK) calibrated to the manufacturer’s instructions. The measurement followed a standard protocol. For each surface, ten measurements were performed over a length of 1.75 mm with a distance of 1.75 mm between them. Mean roughness value (Ra value) was calculated for each surface to document the initial conditions within the groups and to examine the influence of the standardized surface polishing. All specimen preparation steps were carried out by a single experienced operator.

### CAP treatment

**Fig. 1:**
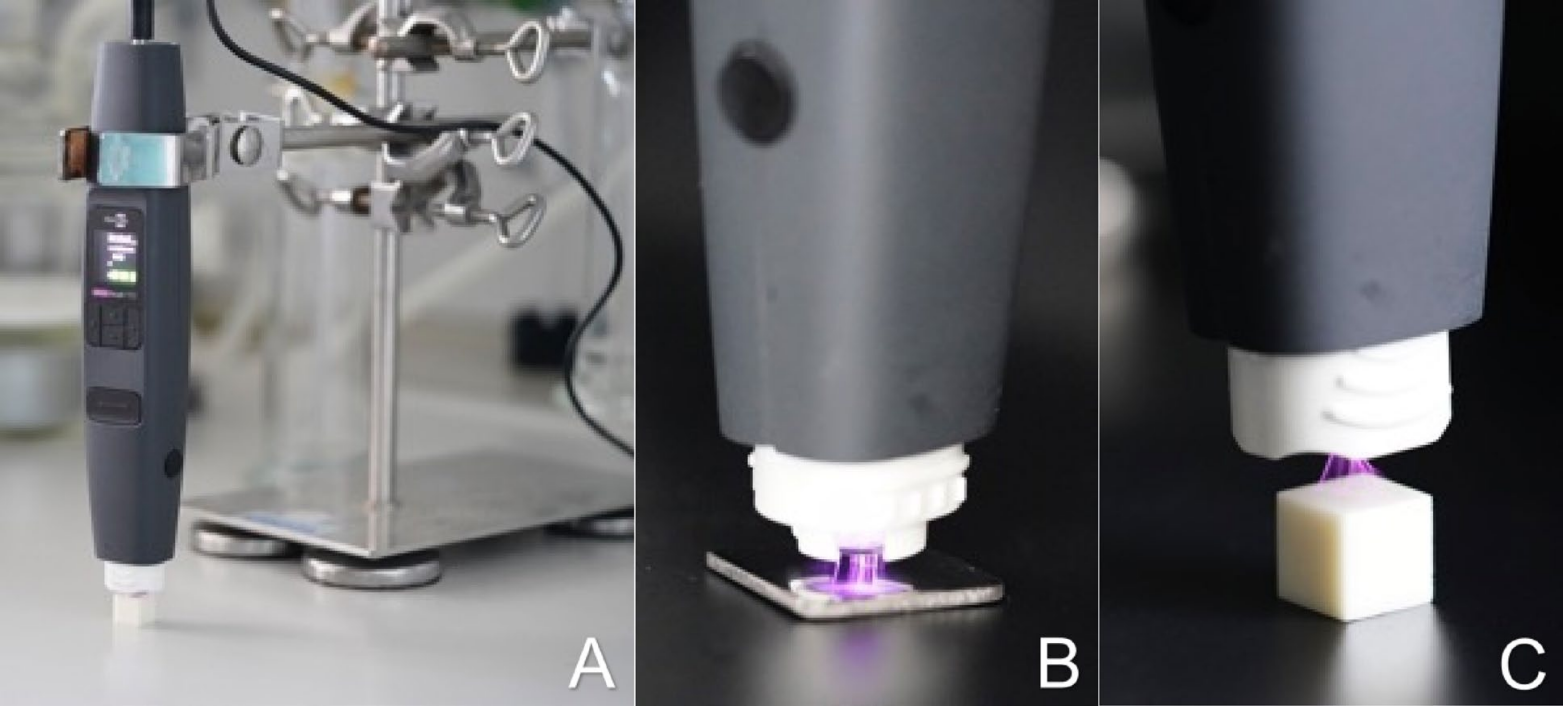
CAP treatment: **A** PZ3 plasma device mounted in the holder, **B** CAP treatment of the titanium surface,**C** CAP treatment of the zirconia surface (distance increased for illustrative purposes)

The specimens of each material were divided into 5 groups (each *n* = 15) according to the CAP exposure time. The 5 groups included a control group (no CAP treatment) and groups with 5-, 10-, 20- and 30-seconds CAP treatment with maximum power (max. plasma and substrate temperature 50 °C; input voltage 230 V; operating power 18.0 W). The “Piezo-Brush^®^” PZ3 hand-held device (Relyon Plasma GmbH, Regensburg, Germany) was used as the CAP source. The CAP-device uses ambient air as process gas. To ensure a constant distance between the material and the nozzle, the device was attached to a holder. For the CAP treatment of the titanium specimens, the nearfield nozzle for conductive materials and for the ceramic specimens needle nozzle for non-conductive materials was selected with a working distance of 0.5 mm for titanium and 2 mm for ceramic materials (Fig. 1).

### Contact angle measurement

**Fig. 2:**
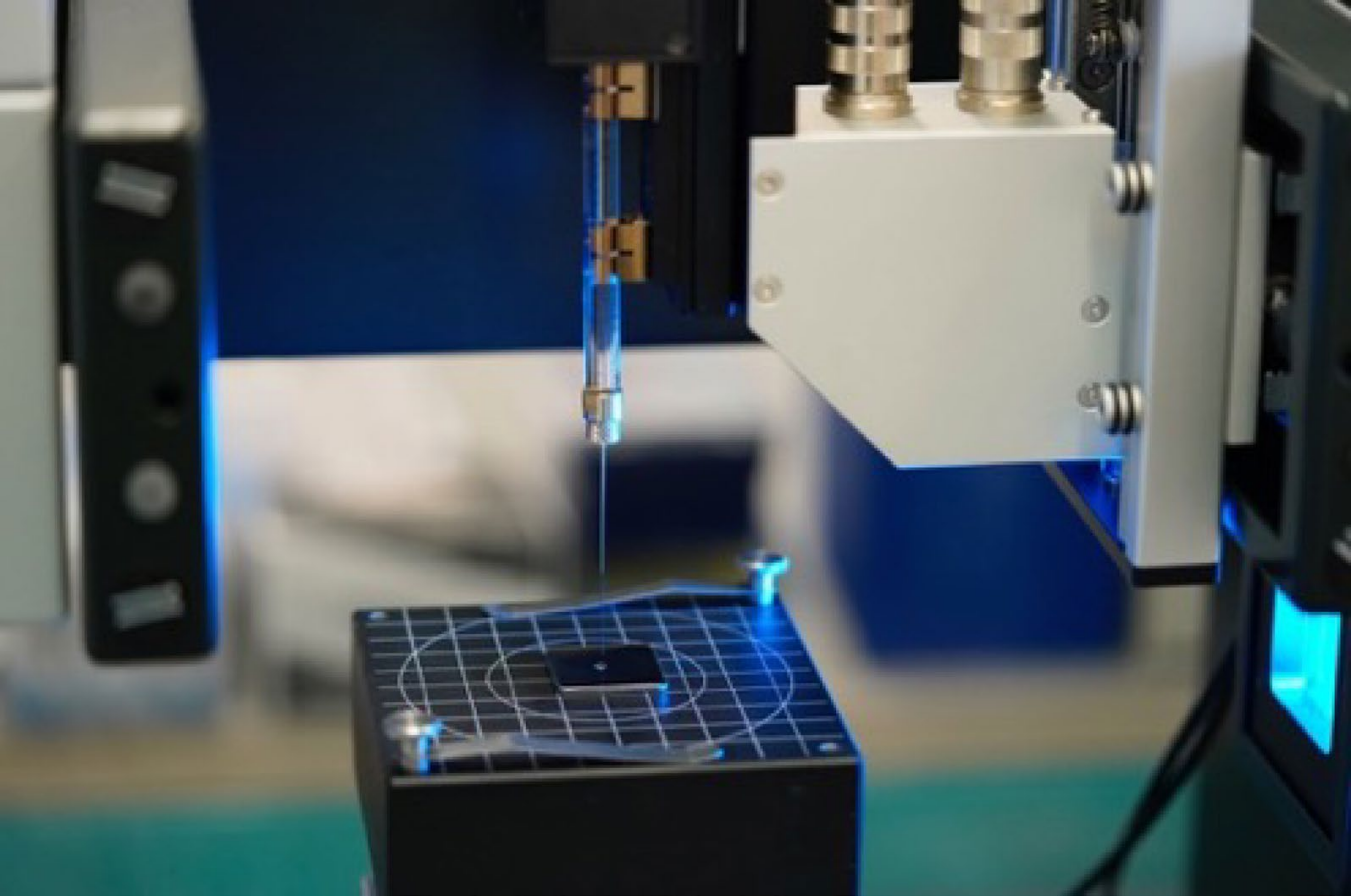
Goniometer used for CA measurement

The CA measurements were carried out at the Max Planck Institute for Polymer Research (Mainz, Germany). A goniometer (Drop Shape Analyzer DSA100E, KRÜSS GmbH, Hamburg, Germany) was used to determine the CA of the materials. Distilled water was drawn into a 0.1 ml syringe and a 3 µL drop of water was applied to the surface at a rate of 1 µL/sec. The CA measurements were carried out immediately after the CAP treatment (Fig. 2). The goniometer provides CA measurement for each drop at the left and right side. With the goniometer, the values were generated by evaluating a series of images using the system-software (KRÜSS ADVANCE 1.9.2.2, KRÜSS GmbH, Hamburg, Germany). All measurements were performed under constant parameter settings.

### Statistical analysis

Data processing and statistical analyses were performed using Microsoft Excel (version 16.82) and IBM SPSS Statistics (version 23; IBM Corp., Armonk, NY, USA). To compare mean CA at different CAP exposure times, one-way analysis of variance (ANOVA) was applied when the assumption of normality was met, as assessed by the Shapiro–Wilk test. If not, the Kruskal–Wallis test was used as a non-parametric alternative. To control the cumulative type I error associated with multiple pairwise comparisons, a Bonferroni-corrected post hoc test was applied, with the significance level adjusted to *p* = 0.005.

## Results

Table 1 presents the surface roughness measurements (Ra) of the investigated materials. PICN showed the highest mean surface roughness (1.85 ± 0.18 μm), followed by zirconia (0.60 ± 0.05 μm) and titanium (0.45 ± 0.12 μm), while lithium disilicate exhibited the lowest mean roughness (0.25 ± 0.05 μm).

**Table 1:** Mean surface roughness values (Ra) for each material in µm

Material	*n*	Minimum (µm)	Maximum (µm)	Mean (µm)	Standard deviation
Titanium	75	0.23	0.71	0.45	± 0.12
Zirconia	75	0.53	0.75	0.60	± 0.05
Lithium disilicate	75	0.14	0.38	0.25	± 0.05
PICN	75	1.60	2.30	1.85	± 0.18

### Contact angle

**Fig. 3:**
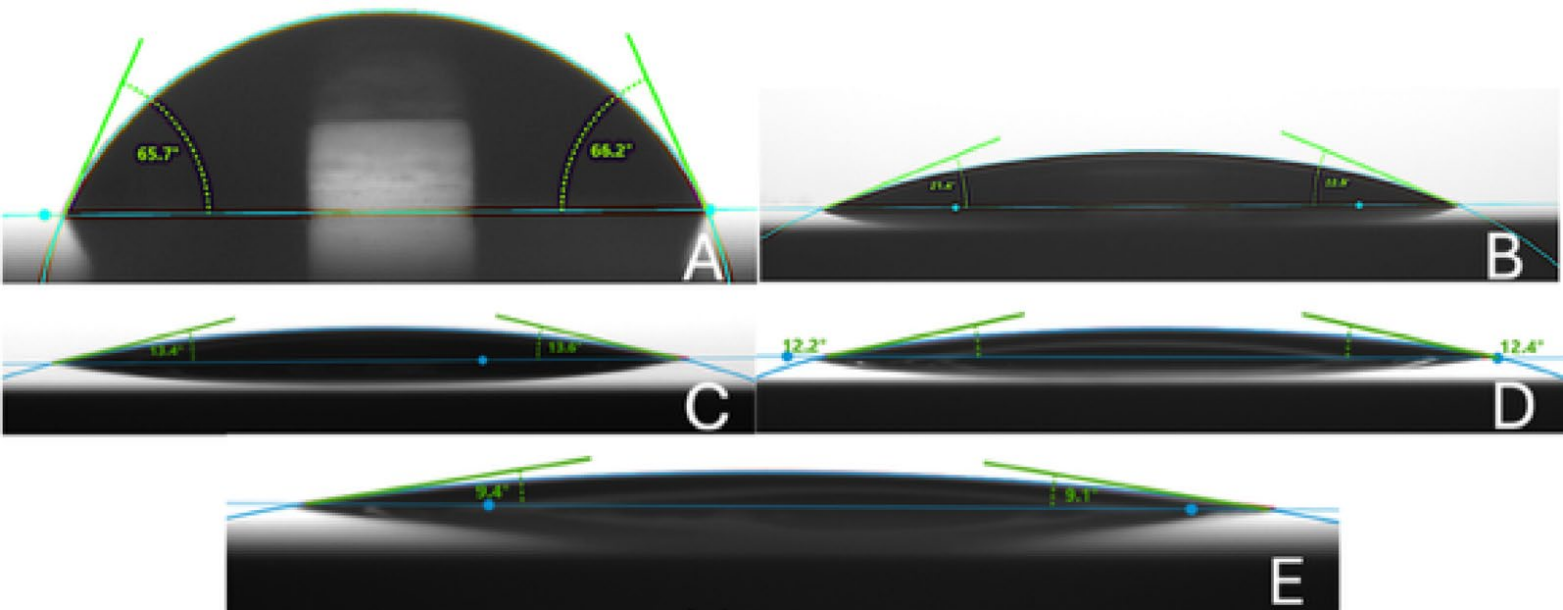
Contact angle (CA) of a 3 µL water droplet on a titanium surface after CAP exposure for **A** 0 s, **B** 5 s, **C** 10 s, **D** 20 s, and **E** 30 s.

**Fig. 4:**
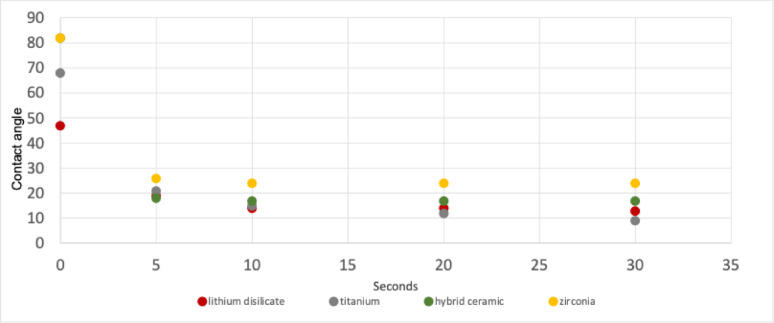
CA measurements on all samples at different CAP exposure times

All investigated materials exhibited a significant decrease in CA after 5 s of CAP exposure (Fig. 3; Tables 2 and 3; *p* < 0.001). Significant reduction of CA beyond 5 s of CAP treatment was only observed for titanium and lithium disilicate. In both materials the 10 s CAP treatment achieved a significant reduction in CA compared to 5 s treatment (titanium *p* < 0.001, lithium disilicate *p* = 0.003). For lithium disilicate, no further significant reduction of CA was observed with (Fig. 4) extended CAP treatment beyond 10 s (*p* = 1.000), whereas for titanium, an additional reduction compared to 10 s was achieved when the exposure time was increased to 30 s (*p* < 0.001).

For zirconia and PICN surfaces, prolonging the CAP exposure beyond 5 s did not result in any additional significant reduction in CA (Tables 2 and 3; *p* = 1).

**Table 2:** Contact angle values (°) of different materials after CAP treatment. Values are presented as mean ± standard deviation (minimum–maximum)

Material	0 s	5 s	10 s	20 s	30 s
Titanium *N* = 15	68° ± 4° (64°–75°)	21° ± 3° (17°–26°)	15° ± 3° (9°–19°)	12° ± 2° (10°–16°)	9° ± 1° (7°–11°)
Zirconia *N* = 15	82° ± 10° (61°–95°)	26° ± 3° (22°–33°)	24° ± 3° (19°–29°)	24° ± 3° (20°–29°)	24° ± 3° (19°–30°)
Lithium disilicate *N* = 15	47° ± 7° (35°–58°)	19° ± 3° (16°–25°)	14° ± 2° (12°–18°)	14° ± 2° (9°–17°)	13° ± 2° (10°–17°)
PICN *N* = 15	82° ± 10° (61°–93°)	18° ± 3° (14°–24°)	17° ± 3° (13°–24°)	17° ± 3° (12°–23°)	17° ± 3° (10°–21°)

**Table 3:** Bonferroni-adjusted p-values for pairwise comparisons of CAP exposure times (significance level adjusted *p* = 0.005)

Comparison (s)	Titanium	Zirconia	Lithium disilicate	PICN
0 vs. 5	< 0.001	< 0.001	< 0.001	< 0.001
5 vs. 10	< 0.001	1.000	0.003	1.000
5 vs. 20	< 0.001	1.000	< 0.001	1.000
5 vs. 30	< 0.001	1.000	< 0.001	1.000
10 vs. 20	0.072	1.000	1.000	1.000
10 vs. 30	< 0.001	1.000	1.000	1.000
20 vs. 30	0.015	1.000	1.000	1.000

## Discussion

The aim of this study was to investigate the influence of CAP on the wettability of the dental materials titanium, zirconia, lithium disilicate ceramic and polymer-infiltrated hybrid ceramic network material measured by CA. For all materials, a significant drop in CA was detected after 5 s of CAP treatment with the device used in the present investigation. Therefore, the null hypothesis, that CAP treatment would not affect the wettability, was rejected. In the titanium and lithium disilicate specimens, the CA was further reduced by prolonged CAP treatment at 30 s for titanium and 10 s for lithium disilicate. Zirconia and PICN showed no further reduction in CA, increasing CAP exposure time to more than 5 s.

In the present study, common materials for single implant crowns were selected. In comparable studies, primarily zirconia and titanium were examined [24–28]. Furthermore, there are studies that have focused on alternative materials such as feldspar ceramics, polymethyl methacrylate (PMMA), or leucite ceramics, which have reported an improvement in bond strength after plasma treatment [29–31]. Jassim et al. used the same CAP device (PiezoBrush^®^ PZ3, Relyon Plasma) as in the present study. However, CAP exposure time and nozzle–surface distance differed [32]. In their study, CAP treatment for 80 s at a greater distance resulted in CA of 7° for zirconia. The present study achieved CA of 24° with no further reduction after 10 s at a reduced distance. These findings indicate that plasma efficiency is strongly influenced by its application parameters, even when the same device is used. Jassim et al. also investigated the relationship between plasma treatment and bond strength, finding an improvement of adhesion without altering the surface as it occurs while sandblasting with aluminum oxide [32]. In another study by Silva et al., the CA of titanium and zirconia specimens were examined after 5, 10 and 20 s of pretreatment with non-thermal plasma and atmospheric pressure. They demonstrated that a 10 s plasma exposure resulted in a decrease of CA from15° to 0°. In contrast to the present study, an MDP primer was used for specimen preparation instead of distilled water [28]. Therefore, the smaller CA could also be attributed to the MDP primer. In a study by Akram et al., titanium got pretreated with atmospheric pressure plasma after sandblasting. The CA of water decreased with the duration of the sandblasting treatment to a minimum of 25° after 120 s and the surface energy increased with prolonged CAP exposure time. CA decreased to a minimum of 8.1° after 15 min of plasma exposure with a distance of 10 mm [25].

In the present study, standardized polishing and cleaning procedures resulted in reproducible Ra values within each material group. This was performed to prevent the surface roughness having a substantial influence.Other studies investigating the influence of plasma treatment on CA are not directly comparable to the present study because different polishing and cleaning methods were performed [33]. Another difference occurs in studies that used cylindrical shaped specimens instead of flat ones [34, 35]. The cylindrical design is more comparable to the actual geometry of an abutment or an implant, but it does not allow a direct comparison with the flat surfaces of other parts. In addition, the round surface can have an influence on the CA of the liquid droplet.

In the present study, a holder was used to ensure a constant distance between the plasma device and the surface of the specimens. The applied working distances were selected in accordance with the manufacturer’s recommendations, which specify not only different nozzle types but also different minimum working distance ranges for electrically conductive and non-conductive materials. For conductive materials, a minimum working distance of 0.5 mm is recommended, whereas for non-conductive materials, a minimum distance of 2 mm is advised.

Accordingly, titanium specimens (conductive) were treated using the near-field nozzle at a distance of 0.5 mm, while ceramic materials (non-conductive) were treated using the needle nozzle at a distance of 2 mm. In both cases, the shortest recommended distance was deliberately selected to ensure maximum plasma efficiency and standardized application conditions. Korzec et al. were able to prove that the size of the effectively activated area depends on the distance between the plasma nozzle and the material [36]. Jassim et al., who also conducted their investigation using a “Piezo-Brush^®^” PZ3 and employed a plasma activation time of 80 s, worked at a distance of 5 mm. This differs from the distance used in the present study and from the recommendations of Korzec et al. [33, 35]. Consequently, as plasma effectiveness is distance-dependent, the use of different nozzle–surface distances and nozzle types may have influenced the results and limits direct comparability between materials.

In some other studies investigating the influence of plasma on wettability, other process gases such as pure oxygen, argon or mixtures of these two, were used. In these studies, no greater differences in the reduction of the CA were observed compared to the present study working with ambient air [24, 28]. The use of ambient air does not appear to be a disadvantage of the method used here, especially given that this procedure is easier to carry out and requires less technical equipment.

Several limitations of the present study should be acknowledged. First, only surface wettability, assessed by CA measurements, was evaluated. Although wettability is an important parameter for bonding, adhesive performance is multifactorial and influenced by factors such as surface chemistry, surface energy, roughness, and interactions with primer or bonding systems. Second, the results are limited to the specific experimental setup, including the plasma device, treatment parameters, nozzle–surface distance, and ambient conditions, which may affect plasma efficacy and limit comparability with other studies. Third, flat, standardized specimens were used, which do not fully reflect clinical geometries. Finally, only a limited range of dental materials was investigated, restricting the generalizability of the findings.

One factor that may potentially influence the bond strength of an adhesive system could be the humidity. Variations in ambient temperature and humidity may affect the consistency of CAP application and should be evaluated in further studies. Although CAP treatment successfully reduced the CA, its clinical relevance and potential to enhance bond strength remain questionable. Previous studies using the same CAP showed no improvement in the bond strength of zirconia crowns to titanium bases after application to zirconia for 15 s and titanium bases for ≥ 30 Sect [18]., nor after 30 s application to PICN crowns compared with conventional conditioning [19]. In both cases, bond strength was reduced. Successful adhesive bonding requires intimate interfacial contact, which is strongly influenced by surface wettability. Based on the CA, wetting is classified as non-wetting (CA > 90°), wetting (CA < 90°) and spreading (CA ∼ 0°). Optimal conditions promote spreading without excessively reducing liquid surface tension and thereby impairing cohesive forces [3]. The reduced pull-off values observed after CAP treatment in previous studies [22, 23] may be attributed to excessive CAP exposure, leading to an over-reduction of surface tension and weakened cohesive forces within composite system. Further studies are required to evaluate alternative primer/bonder systems, different ceramics materials, and the influence of humidity.

## Conclusion

Cold atmospheric pressure plasma treatment significantly improves the surface wettability of titanium, zirconia, lithium disilicate ceramics and polymer infiltrated hybrid ceramic commonly used in dentistry. A reduction in the contact angle was observed for all materials after 5 s of treatment. Within the limitations of this in vitro-study, prolonged cold atmospheric pressure plasma exposure did not result in further reduction of contact angle. Further studies are required to evaluate the optimal application parameters and to determine the potential impact on bond strength.

## Data Availability

The datasets used and/or analyzed during the current study are available from the corresponding author on reasonable request.

## Abbreviations

CA: Contact angle
CAD: Computer-aided-design
CAM: Computer-aided-manufacturing
CAP: Cold atmospheric-pressure plasma
Sec: Seconds
µm: Micrometer
µL: Microliter
µSBS: Microshear bond strength
